# Synthetic Corpus Generation for Deep Learning-Based Translation of Spanish Sign Language

**DOI:** 10.3390/s24051472

**Published:** 2024-02-24

**Authors:** Marina Perea-Trigo, Celia Botella-López, Miguel Ángel Martínez-del-Amor, Juan Antonio Álvarez-García, Luis Miguel Soria-Morillo, Juan José Vegas-Olmos

**Affiliations:** 1 Department of Languages and Computer Systems, Universidad de Sevilla, 41012 Sevilla, Spain; 2 Department of Computer Science and Artificial Intelligence, Universidad de Sevilla, 41012 Sevilla, Spain; celbotlop@alum.us.es (C.B.-L.);; 3 SCORE Lab, I3US, Universidad de Sevilla, 41012 Sevilla, Spain; 4 NVIDIA Corporation, Ltd., Hermon Building, Yokneam 20692, Israel; juanj@nvidia.com

**Keywords:** sign language, sign language translation, sign language production, synthetic corpus, neural machine translation, gloss

## Abstract

Sign language serves as the primary mode of communication for the deaf community. With technological advancements, it is crucial to develop systems capable of enhancing communication between deaf and hearing individuals. This paper reviews recent state-of-the-art methods in sign language recognition, translation, and production. Additionally, we introduce a rule-based system, called *ruLSE*, for generating synthetic datasets in Spanish Sign Language. To check the usefulness of these datasets, we conduct experiments with two state-of-the-art models based on Transformers, MarianMT and Transformer-STMC. In general, we observe that the former achieves better results (+3.7 points in the BLEU-4 metric) although the latter is up to four times faster. Furthermore, the use of pre-trained word embeddings in Spanish enhances results. The rule-based system demonstrates superior performance and efficiency compared to Transformer models in Sign Language Production tasks. Lastly, we contribute to the state of the art by releasing the generated synthetic dataset in Spanish named *synLSE*.

## 1. Introduction

According to the World Health Organization (WHO) [1], about 5% of the world’s population (460 million people) has disabling hearing loss. Sign Languages (SLs) are the principal medium of communication for the deaf community, with around 300 different Sign Languages worldwide [2], which only 1% of the population (almost all deaf people themselves and their families) understand. The immediate consequence is a group with difficulties in everyday communication with other hearing people, making access to education, health care, employment, entertainment and social interactions, in which communication plays a major role, more challenging for them.

Therefore, providing a system capable of translating spoken languages into Sign Languages, and vice versa, to facilitate the exchange of information between the deaf community and the rest of the population remains a developing challenge, and technology is present to provide that help (related research projects include https://signon-project.eu, accessed on 20 February 2024, and https://www.project-easier.eu, accessed on 20 February 2024) where mobile devices and wearables [3,4,5] play a fundamental role as they are part of our daily lives. Since SLs are visual languages, they include non-manual features (facial and body expressions) beyond the manual gesture itself to provide additional information. Sign Languages have their own grammatical rules and are developed independently of spoken languages [6], which is why they have such a low rate of comprehension for those who do not know the language, with no word-to-word correspondence to the Sign Language.

In general, the challenge to improve communication between the deaf community and hearing people depends on the direction in which the information exchange flows: on the one hand, the problem can be approached in the hearing–deaf (H2D) direction (also known as Sign Language Production) or, on the other hand, in the deaf–hearing (D2H) direction (which involves Sign Language Recognition and Sign Language Translation).

Advances in Deep Learning in recent years have led to improved research in fields like Sign Language Recognition (SLR), which is the process of identifying signs that include manual and non-manual gestures and translating them into one or more glosses (text representation of a sign). SLR draws on both gesture recognition [7] (being a more generic and previously studied problem) and SL linguistics [8] (including sentence composition and sign morphology). The work of Staner et al. [9] was one of the first to introduce SLR, using Hidden Markov models and focusing on hand-elaborated features [10,11]. Subsequently, hand and upper body poses were used for recognition [12,13]. More recently, studies have used LSTMs [14] and 3DCNNs [15,16] because of their ability to represent spatiotemporal data.

However, SLR presents several challenges [17,18] that make it still an open and unsolved problem. First of all, since SLR is a completely visual language that includes facial and body expressions in addition to gestures themselves (non-manual cues play a crucial role in making sense and meaning out of what is being signed [19]), a system with sufficient capability and accuracy to be able to perceive these more subtle differences is required. This is different from other tasks such as gesture or action recognition previously studied. Another important requirement is the need for a large dataset acquired under optimal conditions (no occlusions, illumination conditions, different viewpoints, etc.). Creating an SL dataset to perform recognition tasks is a time-consuming job; in addition, it requires professional interpreters to validate and correctly represent Sign Language information (sentences, dialogues, etc.). It requires the use and availability of tools to complete the sign annotation and segmentation process.

SLR includes two main categories [18]: Isolated Sign Language Recognition (ISLR) and Continuous Sign Language Recognition (CSLR). In ISLR, recognition occurs at the gloss level, i.e., it aims to recognize isolated glosses with a video or an image as input. This task is related to others already studied such as gesture recognition [20,21] or action recognition [22,23]. Due to the lower complexity in isolated glosses, good results are generally achieved, even though it is not useful for facilitating fluent communication.

**Figure 1:**
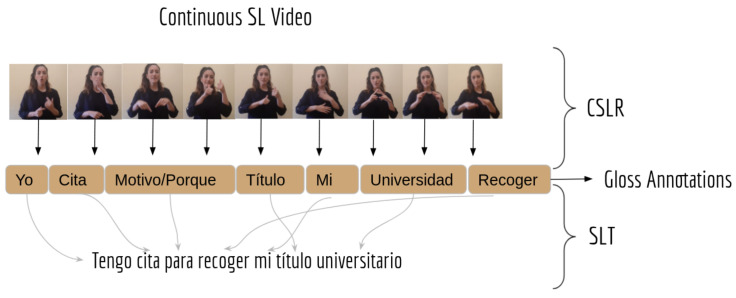
Example of the Continuous SL Recognition (CSLR) and Translation (SLT) process, which receives a video input and produces the translated sentence in a natural language, through gloss annotation (textual representation of glosses). In the example, in Spanish, the translated sentence obtained from SLT is “I have an appointment to get my university degree” which corresponds to gloss annotation, obtained from CSLR, “Me appointment reason/why degree my university get”.

Most SLR research has recently been focused on approaching the problem of CSLR, where the main goal is to recognize each gloss that comprises an SL sentence. The main problem with CSLR lies in the fact that recognizing a sequence of glosses does not usually provide an equivalent sentence or interpretation from the spoken language, as we can see in the gloss annotations in Figure 1. To deal with this problem, recent studies [24] have used Neural Machine Translation (NMT) for Sign Language Translation (SLT), whereby a spoken/written language sentence is constructed from gloss annotations. Thus, with the connection of CSLR with SLT, we can extract the spoken/written language sentence corresponding to a video in which signs are performed continuously [25]. Another work [26] explores an end-to-end solution based on Transformers, from video to written language, but injects the gloss annotation into the model in order to improve results. Glosses have been shown to provide useful information for translation tasks since they provide learning guidance to end-to-end models [24]. However, their annotation is a scarce resource. Works like [27] recognize it as a low-resource NMT task, and propose to use hyperparameter search and back translation. A recent work [28] demonstrates that pre-training with a large synthetic corpus and then fine-tuning with the target, smaller dataset significantly improves the performance of an NMT model for the SLT task on LSE.

Furthermore, there are recent studies [29,30] that try to solve the problem inversely, i.e., instead of providing hearing–deaf communication, they focus on creating a system in which a hearing person can be understood by any deaf person through the generation of videos or a sequence of still images, also known as Sign Language Production (SLP).

The variety of techniques used to facilitate communication with the deaf community through technological advances that have occurred in recent years have been studied in depth, but despite this, no generic and definitive solution has been obtained for this group of people. Instead, results were achieved in specific areas and conditions in different sign languages, among which Spanish Sign Language (LSE, from *Lengua de Signos Española*) is still under study, making it a growing research field where much remains to be done. Moreover, gloss annotations in LSE are very few for machine translation, although there are several ongoing projects making progress, such as *CORLSE* (https://corpuslse.es, accessed on 20 February 2024) and *iSignos* [31].

In this paper, we propose two methods to synthetically create a gloss annotation corpus for LSE, and use them to explore the training hyperparameters of Transformer models. In summary, the main contributions of this study are:

- To provide an overview of Deep Learning-based techniques approached to address the problem of communication between the deaf community and hearing people in the literature, as well as establish the main gaps in recent studies related to these tasks.
- The development of two methods to obtain synthetic gloss annotations in LSE: one based on the translation of an existing dataset (from German to Spanish), and another employing a flexible rule-based system to translate from Oral Spanish (LOE, from *Lengua Oral Española*) to LSE glosses.
- To publish a synthetic corpus including Spanish sentence pairs (LOE) and their corresponding translation to LSE gloss annotations.
- To carry out a set of experiments with language models based on Transformers using our synthetic datasets in both directions: the translation in LOE from LSE to written/oral language (gloss2text) and from written/oral language to glossed sentences (text2gloss).

The rest of this paper is organized as follows: Section 2 provides a context for SL and its main characteristics. in Section 3, we examine the previous research on SLT, SLR and SLP. In Section 4, the synthetic corpus is presented, along with the system to generate it. In Section 5, we introduce NMT together with the models and datasets used in the experimentation. Section 6 describes the protocol to assess accuracy and presents the results. Finally, we conclude the paper in Section 7 by discussing our findings and outlining some possible future work.

## 2. Contextualizing Sign Language

Sign Language is the language through which people with hearing impairment communicate using gestures and visual expressions, and it is independent of spoken languages. There is no universal Sign Language; each country uses its own, sometimes including different dialects. Unlike oral languages, which are based on communication through a mainly vocal–auditory channel, sign languages communicate through a gesture–visual channel; they are also unwritten languages since they have no writing system. Despite this, proposals of transcription systems for sign languages have been developed; however, there are deficiencies in capturing all their communicative features. An example would be glosses, a system of transcription of a sign to text, covering, in addition to manual gestures, body and facial expressions of the signer.

As in any communication channel, there are two elements involved: the sender, which sends the message, and the receiver, which is responsible for receiving it. Communication in SL can occur between deaf people, between a deaf person and an interpreter (a professional who interprets simultaneously from spoken language to SL and vice versa) who acts as an intermediary for the hearing person, and between a deaf person and a hearing person who knows sign language.

In recent years, technology has played an important role trying to make communication between deaf and hearing people who have no knowledge of Sign Language more accessible. Depending on the direction in which communication takes place, different techniques have been developed to improve the exchange of information.

**Figure 2:**
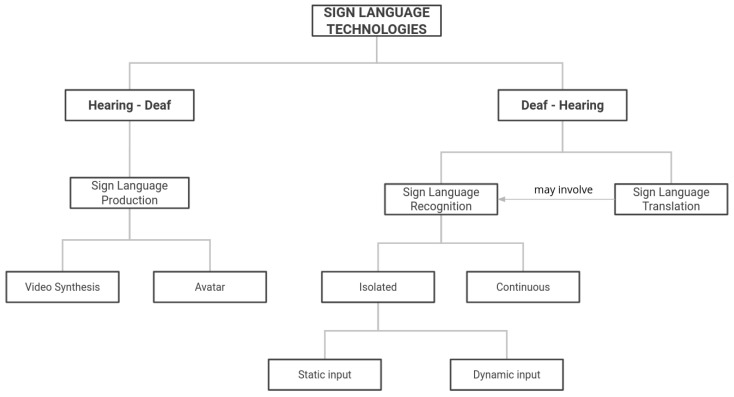
Different computational techniques used for Sign Language communication.

Figure 2 shows the different existing techniques to promote the exchange of information between deaf people and the hearing community. On the one hand, we can represent the signs corresponding to what a hearing person says, for which there are Sign Language Production (SLP) tasks such as video synthesis or avatar generation. On the other hand, there is the recognition and translation of SL to facilitate communication between what a deaf person wants to say and a hearing person. Recognition tasks can be subdivided according to whether they occur in isolation or continuously. In the case of translation, the final objective is to obtain a coherent and meaningful sentence in written language from the input signed sentence.

## 3. Related Work

In this section, we analyze the main techniques and previous work conducted to improve communication between the deaf community and the hearing people.

### 3.1. Towards Deaf–Hearing Communication

In the following subsections, the different methods used to address the problem of communication from sign language to spoken or written language are discussed. For this purpose, two techniques studied in the literature can be distinguished: Sign Language Recognition (as we saw, it can be divided into continuous or isolated), and Sign Language Translation.

#### 3.1.1. Sign Language Recognition

This subsection explains the main categories into which we can classify Sign Language Recognition, as well as an overview of the architectures applied to address this problem. Sign Language Recognition is the task in which glosses made by a signer or a deaf person are inferred from videos or images, subdividing recognition into two categories [18]: ISLR and CSLR.

ISLR shares a lot of features with action recognition, and consequently there are several works using CNNs for feature extraction and classification [32,33,34,35]. Recent work has also relied on employing 3D-CNNs [36,37] to capture spatiotemporal information in an ensemble way. In [38,39,40], an Inflated 3D ConvNet (I3D) architecture [22] is proposed, whose application produces significant improvements in ISLR performance. Pu et al. [41] present an architecture for the ISLR task based on Convolutional 3D networks (C3D) [42] and a Support Vector Machine (SVM) [43] classifier. Hierarchical I3D is introduced for Sign spotting (localize and identify) in a continuous video in [44]. Eunice et al. [45] also use Transformer models for Isolated Sign Language Recognition, increasing the model’s precision by integrating key frame extraction, augmentation, and pose estimation. Skeleton-based architectures [46] have also been used for ISLR tasks.

Moreover, continuous SLR is a more challenging issue, since it must recognize not only the words, but also their correct order and the overall meaning that they provide. One of the principal challenges when applying CSLR techniques is video segmentation and annotation. Each video segment contains a single sign, and since a proper labeling tool is required, the support of a professional interpreter to validate the annotations is necessary. Most architectures used so far usually combine 2D or 3D convolutional networks with temporal models, especially using HMMs, Connectionist Temporal Classification (CTC) [47,48] or LSTMs [49,50]. Compared to 2DCNNs, 3DCNNs learn spatiotemporal features directly through frame sequences.

Spatiotemporal Convolutional 3D networks (C3D) [42] have been previously used in action recognition tasks, introduced by [51] for CSLR. In another work [47], the authors combine a 3D residual convolutional network architecture for feature extraction with stacked dilated CNN instead of LSTM, and CTC for sequence mapping and decoding. In [52], Camgoz et al. introduce a depth-based approach called SubUNets relying on a 2DCNN-LSTM architecture that processes video frames independently, thus improving the learning procedure of intermediate representations. Cui et al. [53] develop a model formed by the combination of CNN and Bi-LSTM. The CNN module is used to capture the fine-grained dependencies, while the Bi-LSTM module captures the information between the gloss segments. Model performance improves with iterative training.

#### 3.1.2. Sign Language Translation (SLT)

Below, we discuss the Sign Language Translation issue by reviewing the different techniques that are applied to address this problem as well as the previous work related to it.

The Sign Language Translation task aims to extract a spoken/written language sentence from a video in which signs are continuously performed. Most recent work has focused on recognizing the sequence of sign glosses that form the CSLR, whose main problem is that it does not provide a meaningful interpretation of what a signer actually says, since sign languages do not share the grammar and linguistic properties of spoken/written language. Because word-to-sign mapping does not exist, the task of translation is of interest.

Recently, Camgoz et al. [25] introduced SLT in two sets of experiments. The first one is based on an end-to-end SLT architecture which aims to translate sign videos into spoken language sentences without any intermediate representation through attention-based NMT models [54,55] instead of just recognizing each sign. The second set of experiments first recognizes the continuous sign video glosses using the CNN-RNN-HMM hybrid method [56], which served as a tokenization layer, and then the spoken language sentences are generated through an attention-based NMT network [54].

In later work, Camgoz et al. [26] presents a method where they train a Transformer encoder using gloss annotations as input, which helps the network to learn more meaningful spatiotemporal representations of the sign by not limiting the information transmitted to the decoder.

Transformers are Deep Learning models that adopt an encoder-decoder architecture to transform one sequence into another. Its main distinction with respect to traditional Seq2Seq models is the absence of recurrent networks (GRU, LSTM, etc.) and the adoption of an attention mechanism in its architecture. The use of self-attention layers over recurrent and convolutional layers is motivated by three factors: the total computational complexity per layer, the amount of computation that can be parallelized, as measured by the minimum number of sequential operations required, and the path length between long-range dependencies in the network.

Yin et al. [57] outperforms Camgoz et al. [26] in the state of the art on the PHOENIX-2014T dataset with their STMC-Transformer architecture, consisting of two networks. The first Spatial-Temporal Multi-Cue (STMC) network is responsible of performing the SLR, and it consists in a spatial multi-cue module that decomposes the input video into its spatial features and a temporal multi-cue module that estimates the temporal correlations at different time steps. The second network of the architecture is a two-layer Transformer [58] network that performs translation from gloss to text.

A different approach is applied in [59], which uses human keypoints instead of SLR techniques as an intermediate step to extract glosses. This set of extracted glosses is used as input to a Seq2Seq model for translation. Kim et al. [60] proposes a method for SLT without gloss annotations, which is also based on the use of keypoints to identify movements while avoiding background noise from an attention-based model.

A detailed survey of SLT techniques is available in [24], concluding that existing datasets are very limited and that glosses provide useful information for training end-to-end (from video to written text) models since they guide mapping frames to glosses. The authors also discuss that larger datasets and better models are needed for SLT.

Regarding this concern, Chiruzzo et al. [28] show that pre-training a language model for SLT with a large synthetic parallel LSE-Spanish corpus, and later fine-tuning with a target parallel corpus, results in outperformance of the behavior of the model. This technique is even better than just adding linguistic information to the dataset, although this also helps.

The authors generated a synthetic corpus using a simple system based on 10 rules to perform translation from LOE to LSE glosses. The system was run over the Ancora corpus, providing 17 k sentences with 500 k words and 400 k synthetic glosses. However, this corpus is not published. The pre-trained model, based on LSTMs with general attention, was fine-tuned over the *ID/DL* dataset [61]. This corpus contains 416 parallel LOE-LSE gloss utterances on a national identity card renewal domain. This work was improved in [62] by adding part-of-speech tags to the dataset. The trained models are also based on LSTM with attention, but the target dataset is *iSignos* [31]. It contains videos from 12 unique signers in several dialogue settings, featuring 2.7 k utterances, 16.5 k words and 7.5 k glosses.

Table 1 shows the different techniques used by previous studies to perform sign language translation tasks. It shows the datasets used, the model or architecture used, whether Gloss2Text or Sign2Text translation is conducted and the result obtained for BLEU-4 reference measure.

### 3.2. Towards Hearing–Deaf Communication

As in the previous subsection, in this one, the research area for the problem of communication in the sense of written/oral language towards sign language is approached. The automatic generation of a video sign language translation given text or speech (from hearing to deaf people) is the task known as Sign Language Production. This area of research is less explored than previous tasks. As a final product, videos are synthesized from each gloss or, alternatively, avatars are generated to sign the corresponding SL [64,65].

#### 3.2.1. Sign Language Production (SLP) with Videos

Previous work related to Sign Language Production through videos is explained and developed below.

Generating videos of humans performing glosses is a difficult task due to the need for consistent frames to show coherent motion, in addition to the complexity and variety of actions that can occur in these videos. The field of automatic video generation has undergone great advances due to the use of neural network-based architectures such as GANs [66] or RNNs [67].

Among previous works that stand out, San-Segundo et al. [61,68] propose to translate Spanish Speech into Spanish Sign Language through four modules: speech recognizer, semantic analysis, gesture sequence generation and gesture playing. The main drawback of this work is that the system is limited to the comprehension of the manual alphabet, i.e., word-level signs.

In [69], a new methodology is introduced for the speech-to-SLT problem, in which the authors divide the task into three stages: translation from text/speech to gloss, prediction from text/gloss to skeleton, and skeleton to video synthesis. For this purpose, they also introduce the How2Sign dataset, which is also used in [70], based on the *Everybody Dance Now* approach [71], generating videos of a signer given a set of keypoints. The model is trained with this dataset, which extracts the keypoints from the input video, and these are used to generate video frames through a Generative Adversarial Network.

In [72], sign language video sequences are generated through a generative model using pose information resulting from data-driven mapping between glosses and skeletal sequences. Subsequently, Stoll et al. [73] present a two-stage SLP model: first, they combine a Neural Machine Translation (NMT) network with a Motion Graph, translating spoken sentences into sign pose sequences, which are used by a second stage of Generative Adversarial Networks (GANs) that produce photo-realistic sign language video sequences.

Another recent work is [74], which proposes an end-to-end model that translates spoken sentences into continuous 3D sign sequences. The model is based on a progressive Transformer-based architecture, which formalizes a Symbolic Transformer architecture that converts a spoken sentence into a gloss representation as an intermediate step and then applies the Progressive Transformer architecture which converts the symbolic domains of gloss or text into continuous pose sequences. Similar to this work is the paper of Zelinka et al. [75], in which the authors focus on a fully end-to-end automatic text-to-video Sign Language synthesis system based on a feed-forward Transformer and a recurrent Transformer.

#### 3.2.2. Sign Language Production with Avatars

In this last section of the related work, SLP is studied, focusing, in this case, on the generation of avatars.

The necessity of interpreters as intermediaries to support communication between hearing and hearing-impaired people is a costly solution in many areas of everyday life. That is why offering a similar solution in certain contexts is something that researchers are working on. The use of avatars as a technique for displaying signed conversations through 3D animated models is one such solution. In this context, there are already projects that use avatars to interpret SL, such as HandTalk [76], which provides an API and an app that translates natural language text input into Brazilian SL using an artificial interpreter called Hugo.

Another approach, TESSA [77], tries to translate English speech into British SL, which aims to develop a system under the domain of post office counter service that allows communication with a deaf customer. The system maps the user’s question to a possible sentence through speech recognition, subsequently synthesizing the appropriate sign sequence into BSL. The Voice-Activated Network-Enabled Speech and Sign Assistant (VANESSA) [78] system uses an avatar that provides assistance in British SL between attendants and their deaf clients in a Council Information Center (CIC).

The use of avatars can also present challenges, such as the lack of non-manual information (facial and body expressions) or the presence of unnatural movements which reduce the understanding of what is intended to be communicated in Sign Language [79]. To avoid this, some works have also focused on annotating facial and body information [80,81].

## 4. SynLSE Corpus: A Synthetically Generated Corpus for LSE Translation

In this section, we discuss our proposed synthetic parallel corpus, *SynLSE*, for written LOE (Spanish), and LSE (gloss annotation). It consists of three parts: *tranSPHOENIX*, *ruLSE* and *valLSE*. The former is used to explore some hyperparameters for Transformer models, ruLSE is employed for a complete training of selected models, and *valLSE* is a small set with semi-validated sentences. This corpus and the source code of ruLSE are available at https://github.com/Deepknowledge-US/TAL-IA/tree/main/CORPUS-SynLSE (accessed on 20 February 2024), as part of the TAL.IA project https://github.com/Deepknowledge-US/TAL-IA (accessed on 20 February 2024). In what follows, we depict each of them.

### 4.1. tranSPHOENIX: Translation to Spanish of RTWH-PHOENIX-2014T

This section discusses the procedure performed to generate the first part of our proposed synthetic parallel corpus.

Considering that PHOENIX-2014T is the benchmark dataset in the majority of recent state-of-the-art studies addressing the problem of Sign Language Translation, it is the one we decided to use for our research, excluding others such as How2Sign [82] or CLS [51], which does not provide the intermediate gloss annotations required for our study, and others such as SIGNUM [83], much less extensive in size.

The PHOENIX-2014T dataset is considered to be the first largest and most vocabulary-rich continuous SLT corpus, which includes, for each sign language video, a sign–gloss annotation and its equivalent text German translation. RTWH-PHOENIX-2014T is an extension of the PHOENIX14 corpus, and, instead of being recorded under specific laboratory conditions, the entire set was extracted from the weather forecast broadcast of the German public television station PHOENIX, performed by nine different signers. The corpus consists of a vocabulary of 1066 different signs and German translations with a vocabulary of 2887 different words.

To generate a synthetic corpus for LSE, we first proposed to separately translate into Spanish the subsets (train, test and development) that compose the RTWH-PHOENIX-2014T corpus. Although the structure of the German sign language (GSL), as well as its glosses, does not completely correspond to the Spanish one, we believe that its use to create a synthetic corpus can be a good first approximation. On the one hand, we expect that the translation of the written language part is of good quality. On the other hand, although glosses in GSL and grammatical structure are not the same, their direct translation one by one can be useful to study how translation models behave when restructuring linguistic concepts (in this case, glosses) in written language.

There are still unavailable datasets [84] containing gloss annotations with their natural language translation for SLT tasks, since most of the public ones do not provide this type of data. Therefore, we consider it more appropriate for this study to use the RTWH-PHOENIX-2014T dataset because of its considerably larger size in terms of the amount of data, as well as the gloss annotations together with their natural language translation in order to be able to perform SLT tasks.

### 4.2. ruLSE: A Flexible Rule-Based System to Generate Gloss Annotations

The objective of this subsection consists in explaining the methodology applied for the generation of the rule-based system to generate gloss annotation proposed in our work. For this purpose, we compare our method with the artificial corpus, ASLG-PC12, detail the transformation rules, and finally focus on the methodology for the generation of the corpus.

When a word-to-word translation of glosses in another language is performed into Spanish, the syntactic structure of the sentence does not correspond at all to that of the Spanish sign language. An erroneous glossing made by the direct translation can introduce noise to the models. Therefore, we decide to opt for the creation of a synthetic dataset based on the methodology followed by the ASLG-PC12 project [85]. We improve this methodology, and we name it *ruLSE*.

Similarly, a set of transformation rules is defined and a system capable of reading and applying rules is implemented. The defined transformation rules cover up to B1 level (CEFR) in LSE; the levels are extracted from the books of “Confederación Estatal De Personas Sordas” (CNSE). However, in order to be able to extend and refine the dataset in the future, the system is easily extendable and flexible to admit new rules. So far, approximately **80** rules have been collected, which overcomes the nine rules in state-of-the-art research [28].

#### 4.2.1. Differences with ASLG-PC12

One of the main problems with ASLG-PC12 is that the immense number of sentences with different grammatical structures that can be formulated in a language cannot be covered with a limited number of defined transformation rules. This makes it impossible to deal with all possible input sentences. As a solution to this problem, we propose the formulation of more generic rules that can be applied to a subset of words, without the need to match the entire sentence. In this way, the risk that no rule is satisfied for any given input sentence decreases.

By formulating more basic rules, the number of rules that need to be defined decreases, and redundancy is reduced among those rules that define very similar grammatical structures and whose transformation only affects the set of words they have in common. However, it should be taken into account that a single simple rule may not cover all the transformations that a sentence needs. Therefore, another difference with ASLG-PC12 is that we allow for the system to apply more than one transformation rule, if necessary.

Moreover, the Standford CoreNLP is the tool used in ASLG-PC12 to obtain the input sentence information (lemmas and grammatical categories) with which to search and apply the appropriate rule. However, two problems have been encountered for our purpose: obtaining the lemma of words is not available in Spanish, and the grammatical categories it offers are too generic (UPoS, Universal Part of Speech). To define correct transformation rules in LSE, we need a more precise distinction of words, since not all words with the same universal category follow the same rules. Subsequently, we use Stanza, a Python 3 library developed by Standford based on CoreNLP, which offers a solution to the discussed problems: Spanish lemmas and specific grammatical categories (XPoS) as well as universal ones (UPoS).

Finally, the proposed gloss transformation algorithm is more flexible than ASLG-PC12, able to define a wider variety of transformation rules and thus achieve a result that complies with most of the linguistic properties of LSE. A different gloss notation system is also used, as detailed in next section.

#### 4.2.2. Transformation Rules

Each transformation rule consists of an input and an output structure. The former defines the configuration that a sentence must have to comply with the rule, and the latter indicates the transformation to be applied to the original sentence. These structures are formed by a set of elements defined as <id>_<description>. Each element corresponds to a word; therefore, id is a word identifier, the same in the input and output structure. The identifier matches the positional order of each word in the input structure, but does not have to match the order in which they are placed in the output structure. In addition, description indicates, for each word, one of the following:

- Specific (XPoS) or Universal (UPoS) grammatical category. It is represented in lowercase within the rule structures.
- Textual word or lemma. It is represented in uppercase within the rule structures.

Once transformations are applied to the original sentence, the lemmatization of the transformed uppercase is returned. We recall that lemmas are those simple and autonomous words in the lexicon of a language. The result of the lemmatization corresponds to the glossing. Although most of the glosses coincide with the corresponding Spanish lemma that best resembles the meaning, sometimes, the sign also depends on additional information to be added in the gloss after the lemma separated by a hyphen. For example:

- Plural words are signed, in some cases, by repeating the sign or its classifier several times to indicate abundance or frequency. When a word is plural, it is glossed as follows: <lemma>-PL.
- Proper nouns are usually spelled because they do not have a preset sign. In this case, this is indicated as a spelled word as follows: <syllable>-NP.

Transformation rules can be used for the following purposes:

- Sorting: to change the order of words that comply with the input structure.
- Elimination: to delete words that do not have an associated sign in LSE.
- Insertion: sometimes it is required to insert glosses that do not appear in the input sentence by adding the lemma (capitalized) in the appropriate position within the output structure. The id is set to zero: 0_<lemma>.
- Substitution: A word in the input structure can be modified in the output structure for several reasons: several independent signs are associated with it; it is represented by a different gloss than its lemma; more information has to be provided in addition to its lemma separated by a hyphen (useful for plurals). One example for the last reason is the following: the lemma of the word “PERROS” (dogs) is “PERRO”, but in order to keep the plural information, we can define a rule with input structure 1_nc0p000 and output structure 1_nc0p000-PL, obtaining, as a result, “PERRO-PL” (*nc0p000* is the specific grammatical category of plural nouns).

The <id>_* element can also be used in the input structure. This acts as a regular expression, encompassing the set of words found in the position, if any.

Finally, it is sufficient for a subset of consecutive words in the original sentence to match the input structure of a rule. In this case, the transformation applies only to the matching words, leaving the rest of the sentence intact. In this way, it is possible to apply, sequentially, more than one rule to the same sentence. The order of application of rules influences the result, since a rule is applied on the sentence transformed by the previously applied rules and not on the original sentence. Therefore, rules have an associated priority, where more specific rules have more priority than those more generic. Moreover, some rules can be configured so that they are not applied even if they are fulfilled, if another rule with higher priority has been applied previously. These are exclusionary rules, which are defined in our set by a number by which the rules can exclude each other.

Next, we provide a short description of rules with priorities and exclusion that can be applied to a single sentence:

1. Adjectives are signed after nouns.
2. Indirect complements are signed after the verb (Exclusion 1).
3. Direct complements are signed before the verb (Exclusion 1).
4. Articles are not signed.
5. Prepositions are not signed.

Given the above rules, the sentence “El buen perro juega con su dueño” (the good dog plays with its onwer) is transformed as follows: “El buen perro juega con su dueño” $\overset{rule1}{\to }$ “El perro buen juega con su dueño” $\overset{rule2}{\to }$ “El perro buen juega su dueño” $\overset{rule4}{\to }$ “perro buen juega su dueño” $\overset{lemmatization}{\to }$ “PERRO BUENO JUGAR SU DUEÑO”. Rule 3 is not applied.

#### 4.2.3. Parallel Corpus Generation

**Figure 3:**
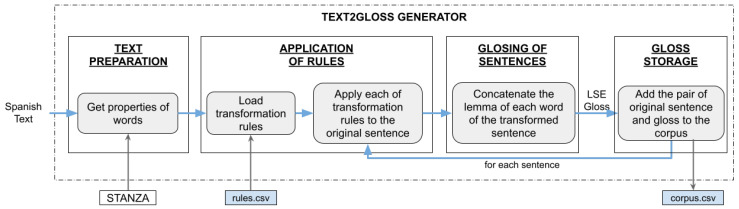
Scheme of the ruLSE algorithm that translates a Spanish sentence into LSE glosses.

Figure 3 shows a scheme of our algorithm, ruLSE, that translates from LOE into glosses in LSE. The four main steps are:

1. *Text preparation*. The algorithm receives a sequence of characters that form a Spanish text. We use the Stanza library to separate the text into sentences by punctuation marks, tokenize the sentences into words, obtain the lemma of the words and syntactically analyze each sentence (providing properties such as the universal and specific grammatical category of each word). At the end, a sentence is a list of words and the text is a list of sentences. Each word has an assigned set of properties according to the aforementioned process.
2. *Application of rules*. The transformation rules are stored in a CSV file. Once loaded, the text phrases are iterated and the transformation rules are applied to each of them. This returns the input sentence but with updated word information: new position in the list of sentences and the new position in the text.
3. *Generation of glosses.* On the transformed sentence, the words are iterated and the lemma of each one is concatenated in the order indicated by its position attribute. This step is repeated for each of the sentences in the text.
4. *Insertion in the corpus*. Finally, each of the sentences of the initial text is inserted together with its glosses in the file where the corpus is stored.

Using ruLSE, we add everyday sentences to our SynLSE parallel corpus, so that the models to be trained on this dataset will be able to transform sentences that are frequently used in our daily life. For this reason, we decide to use the Spanish Speech Text dataset, available at Hugging Face [86]. This dataset offers 403 k sentences extracted from daily conversations, but we generate 10 k pairs using ruLSE. We create three splits of training sets, with 1 k, 3.5 k and 7.5 k pairs.

Since these sentences are more complex than those used in the validation of the ruLSE algorithm, a subset of the generated glosses was reviewed by an expert LSE interpreter. We conclude that the result is not perfect, but quite close to the real one.

### 4.3. valLSE: A Semi-Validated Dataset to Test the Performance of Models

The following section details the dataset used to test the performance of the models used. This dataset contains validated sentences to be used to test and compare the accuracy of the models when translating LOE to/from LSE. It is, in turn, split in two parts:

- *Macarena:* A small set of 50 sentences from the *Large Spanish Corpus* (https://huggingface.co/datasets/large_spanish_corpus, accessed on 20 February 2024), which contains news in Spanish. These sentences were translated to LSE glosses using our ruLSE system, and they were semi-validated by an expert interpreter. This means that only 10 sentences were reviewed, but since the complexity of the sentences is very similar, the interpreter assumed that the same corrections could be applied to the rest. According to her, the translation could be improved but it was correct (see Section 7 for more details).
- *Uvigo:* The LSE_UVIGO dataset [87], as published in 2019 on the authors’ website. Some words were modified to meet the notation followed by ruLSE. Specifically, the changes were the following: rewriting nouns separated by hyphens as *word-NP*; adding PL to plural nouns and removing *(s)*; and removing periods and hyphens between words.

## 5. Experiments on Neural Sign Language Translation

In this section, we introduce the Sign Language Translation models applied in the experimentation, describing the configuration setup and employed datasets. In general, most of the works in the literature on SL focus on gloss recognition by applying the different techniques mentioned above. Therefore, we now center our study on Sign Language Translation. SLT can be approached in two ways: on the one hand, there is a procedure consisting of two stages, where CSLR is first used to convert the video into sequences of glosses and then the translation of the glosses into spoken language is performed [57,88]. On the other hand, the translation of the signed videos into spoken language can be performed without intermediate representation [25,59].

In our case, we approach SLT as a second step after applying CSLR to videos using Neural Machine Translation (NMT) techniques on a dataset containing gloss annotations to obtain translations into written language. This allows us to take the first step in the future towards the SLP described in Section 3.2.1.

### 5.1. Neural Machine Translation

In the following subsection, we review the advances in Neural Machine Translation (NMT) field between languages over the years.

NMT relies on the use of neural networks to perform automatic text translation, where Seq2Seq [89] architectures are particularly well suited to address this problem, and have been successfully used for language translation. Seq2Seq is a neural network that transforms a sequence of input elements into another sequence, and it consists of an encoder that obtains the source sequence and a decoder that produces the target sequence. Early approaches focused on the use of convolutional networks and recurrent networks, in particular LSTM-based models [90] or GRU [91] as recurrent networks when dealing with longer sequences.

Bahdananu et al. [55] introduced the attention mechanism to improve translation performance on long sequences by allowing for the decoder to have additional information about the hidden state of the encoder, avoiding the bottleneck problem caused by standard Seq2Seq networks that are unable to model long-term dependencies in large input sequences. Similarly to the architecture proposed in [55], Luong et al. [54] introduce improvements by using hidden states only in the top of RNN layers.

Vaswani et al. [58] introduce a novel architecture called Transformer in paper “Attention Is All You Need” which drastically improves translation performance over other architectures, making them the state-of-the-art model [92] for NMT tasks. As the title indicates, it uses the attention mechanism, which is used to decide which elements of the input sequence are relevant.

Classical neural machine translation method starts by embedding the input and output tokens, which in our case would be the sequence of glosses of the source sentence and the sequence of words in the target spoken language. The main purpose of using word embeddings is the transformation of each word in the set into vector representations, so that all words are equidistant from each other. In [93], Qi et al. demonstrate that using pre-trained word embeddings in source or target languages helps to increase evaluation metric scores, so different pre-trained embeddings in Spanish are used for our experiments.

Yin et al. [57] propose a two-module architecture for video-to-text translation which outperforms the state of the art on gloss-to-text and video-to-text translations. For this purpose, the first STMC (Spatial-Temporal Multi-Cue) module performs the task of CSLR from videos, while the second module is composed of a Transformer network that performs the translation of the sequence of sign glosses obtained as output of the CSLR module into written/spoken language.

### 5.2. Transformer Models for SLT Experiments

The purpose of this section is to specify the models for sign language translation that we use for the experimentation proposed in our method.

For the set of experiments, two different models were used: STMC-Transformer and the MarianMT model, both based on the original Transformer of Vaswani et al. [58]. On the one hand, STMC-Transformer was relying based on the experimentation carried out in [57]. This model is referenced in the original Transformer paper proposed by Vaswani et al., whose architecture details are maintained except for the number of encoder–decoder layers used, which is shown in Section 6. On the other hand, the MarianMT model was also derived from the “base” model of Vaswani et al., but in this case, it was originally trained using the Marian C++ library [94], which allows fast training and translation.

In summary, this set of experiments was organized into four groups:

1. **Text2gloss on the original PHOENIX-2014T dataset**. The STMC-Transfomer model was trained on the original (German) version of PHOENIX-2014T dataset to find the optimal number of layers in the model. We explored different numbers of encoder–decoder layers: 1, 2, 4 and 6.
  This proved that the best results are obtained for a 2-layer configuration in the encoder and in the decoder of the Transformer; therefore, this was the number of layers used in the remaining experiments for this model. This Transformer was configured with word embedding size 512, gloss level tokenization, sinusoidal positional encoding, 2.048 hidden units and 8 heads, and for optimization, Adam was applied. The network was trained with a batch size of 2048 and an initial learning rate of 1; 0.1 dropout, 0.1 label smoothing.
2. **Text2gloss on tranSPHOENIX dataset (from SynLSE)**. In the second block of experiments, we trained both models (STMC-Transformer and MarianMT model) on different subsets of tranSPHOENIX: one formed by the whole dataset (7096 sentences in the train set and 642 in the test set), another one formed by approximately half of the dataset (3500 sentences in the train set and 321 in the test set) and the last smaller set formed by 1000 sentences for training and 92 for testing. Thus, based on the results obtained, it was possible to establish the number of sentences in the training set necessary for the model to learn an acceptable translation of glossed sentences into written language.
  The STMC-Transformer was initialized with pre-trained embeddings (i.e., trained in a large corpus of text in the desired language) for transfer learning. Two word embeddings, trained in an unsupervised manner on a large corpus of data in Spanish, were used to improve the experiments on the dataset: GloVe [95] and FastText [96]. The corpora on which they were trained are Spanish Unannotated Corpora (SUC) [97], Spanish Wikipedia (Wiki) [98] and Spanish Billion Word Corpus (SBWC) [99].
  Moreover, the pre-trained MarianMT model from HuggingFace library was fine-tuned for the experiments, using a batch-size of 4, an initial learning rate of $2\times 10^{-5}$ for the Adam optimizer with weight decay fix and 20 epochs.
3. **Text2gloss on ruLSE dataset (from SynLSE)**. A third group of experiments focused on training the best STMC model from the previous group and MarianMT, and their performance was tested when trained on the more accurate, larger and synthetic dataset generated with ruLSE. We also tested the performance of the best model towards the valLSE dataset, and compared it with our ruLSE system.
  MarianMT was trained with 1000, 3500 and 7500 sentences from the parallel corpus generated with ruLSE (ruLSE dataset). A hyperparameter search was performed for each subset, and the here reported results were achieved using 1000, 3500 and 7500 sentences of the dataset. The best training performance for 1000 sentences was achieved with a learning rate of $5.97\times 10^{-5}$, 10 epochs, a batch size of 16 and weight rate 0.076; a learning rate of $4.85\times 10^{-5}$, 15 epochs, a batch size of 64 and weight rate 0.092 for 2000 sentences; and a learning rate of $6.5\times 10^{-5}$, 10 epochs, a batch size of 32 and weight rate 0.034 for 7500 sentences.
4. **Gloss2text when using ruLSE versus STMC-Transformer and MarianMT**. The last group of experiments consisted of applying the STMC-Transformer and MarianMT models on the entire synLSE corpus but for Sign Language Production. The objective of this experiment set was to test whether Transformers obtain similar performance one way or another, given that glosses can produce several text sentences but text2gloss mapping is unique. This was accomplished by inverting the training data modalities, having as input the sequence of sentences in written language and obtaining as output the sequence of glossed sentences (i.e., gloss production). We tested the performance against our rule-based system ruLSE using our short validated dataset. The employed models and configurations were the same, also regarding the use of previously pre-trained word embeddings.

The computation and memory costs of training were low for the set of experiments run on the STMC-Transformer model (training time between 10 and 15 min), but for the MarianMT model, the execution time increases between 30 and 40 min (depending on the amount of data used). Experiments with tranSPHOENIX were run on an NVIDIA GeForce RTX2060 graphics card installed in a laptop (Sevilla, Spain), which contains 6 GB of memory. Experiments with the ruLSE subset were run on the following graphic cards: an NVIDIA A100 with 48 GB (installed in a HPC server based at CICA, Sevilla, Spain) and on a T4 with 16 GB (accesed on Google colab).

### 5.3. Employed Metrics

Metrics used to determine the performance of the models explained in the previous section are reviewed below.

The most widely employed evaluation metrics for text2gloss (SLT) and gloss2text (SLP) are BLEU [100] and ROUGE [101]. The BLEU [100] metric, which stands for Bi-Lingual Evaluation Understudy, is popularly known in neural machine translation to evaluate translation accuracy. BLEU gained popularity because it was one of the first evaluation metrics for NMT that managed to report high correlation with human evaluation criteria.

BLEU attempts to measure the correspondence between a machine translation output and a set of high-quality reference translations. The BLEU score comprises two parts: the brevity penalty, which penalizes generated translations that are too short compared to the closest reference length with an exponential decrease, and the n-gram overlap, which counts the number of unigrams, bigrams, trigrams and four-grams that match their n-gram counterpart in the reference translations. Specifically, four BLEU-grams ranging from 1 to 4 are used, with BLEU-4 standing out as the most relevant.

The central idea behind BLEU is that the closer a machine translation is to a professional human translation, the better it is. In other words, it tries to measure adequacy and fluency in a similar way to that of a human, aiming to transmit in the output the same meaning as that of the input sentence, the result being good and perceived as fluent in the target language. The BLEU metric scores a translation on a scale between zero and one, with the score closest to one considered the best translation by the system. Our experiment results are shown on a scale from 0 to 100 to make tables and figures more compact and comprehensible.

The ROUGE [101] metric is also for evaluating automatic summarization of texts. In this case, it compares an automatically produced summary or translation against a set of reference human summaries. We use ROUGE L F1 as the evaluation metric in the experiments and refer to it as simply ROUGE.

## 6. Results

This section analyzes the results obtained in each experiment mentioned above. All reported results are averaged over five runs with different random seeds.

### 6.1. Experiment Sets 1 and 2 (text2gloss Based on PHOENIX-2014T)

Table 2 shows the results of Experiment Set 1. As explained in the previous section, the purpose of this first set of experiments is to find the optimal configuration architecture proposed for the rest of the experiments. Since the dataset used is small compared to those typically employed for natural machine translation tasks, it can be seen that a smaller network is advantageous, obtaining the best results with a two-layer configuration in STMC-Transformer for the decoder and the encoder over the original dataset, achieving the highest score for the BLEU-4 reference metric, confirming the finding in [57].

Next, Experiment Set 2 is focused on the translation of glosses into spoken/written language in Spanish (tranSPHOENIX) using STMC-Transformer with different configurations of pre-trained word embeddings, along with the MarianMT model.

Table 3, Table 4 and Table 5 show the results obtained by applying the same models to training sets of different sizes: 1000, 3500 and 7096 sentences, respectively. On the one hand, as expected, we can see that as the size of the training data increases, better results are obtained. The most significant improvement occurs with the whole training data set, as shown in Table 5, with the trend of improvement quite lower when progressing from the training of 1000 sentences to the one of 3500. It is only up to 1.15 points better in reference measure BLEU-4 for the results shown in Table 3. We can conclude that the improvements in the performance of the model depend on the size of the data used during training, but this improvement in the results is not proportional concerning size, since the results obtained in Table 3 and Table 4 are quite similar despite using up to three times more data during training.

Furthermore, it can be confirmed that applying pre-trained FastText from Spanish Unannotated Corpora on the STMC-Transformer model increases performance for BLEU-4 on all data subsets. The MarianMT model is the one that obtains the best results in general for most measures on all data subsets used in the training.

**Figure 4:**
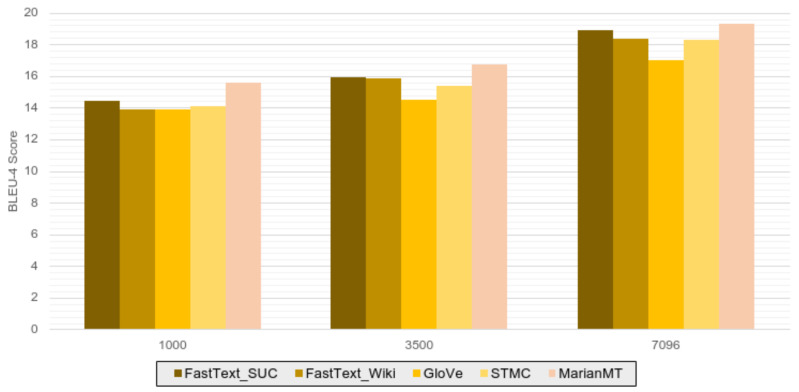
Text2gloss BLEU-4 results obtained on the different subsets of data according to the models and word embeddings used, where 1000, 3500 and 7096 mean the numbers of sentences used for training.

Figure 4 graphically shows the progressive increase in the precision of the results (based on the BLEU-4 reference metric) as the dataset grows, regardless of the use of pre-trained word embeddings in the case of the STMC-Transformer model. The most significant increase occurs using the entire dataset during training. This graph also displays that training the model with a reduced dataset (1000 sentences) does not provide a noticeable difference in the results obtained with different word embeddings, while as the size of the training set increases there is more variance in the performance with different embeddings. It can also be seen that the GloVe algorithm trained on Spanish Billion Word Corpus provides the lowest performance on the STMC model while, on the other hand, it is the MarianMT model that outperforms the other configurations on this model in the results.

**Figure 5:**
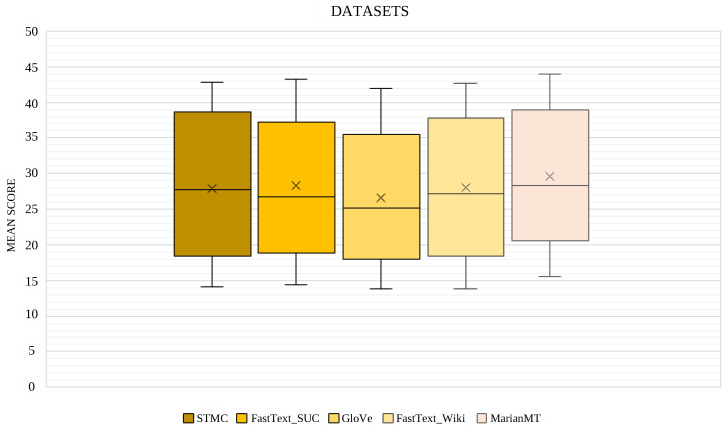
Average score obtained for each of the evaluation metrics used in the different datasets for all the configurations launched in the text2gloss experiments with tranSPHOENIX.

Additionally, Figure 5 shows that employed configurations and models do not generate a large difference in performance between them. Regarding the used models, as shown in the previous tables, MarianMT is the one that obtains better average results compared to the STMC-Transformer model and its different configurations. Distinguishing these configurations, it can be seen that despite obtaining better results by applying pre-trained word embeddings in Spanish, the performance of the model does not always increase. This proves that, using transfer learning through applying GloVe trained on Spanish Billion Word Corpus as pre-trained weights, the lowest measures for BLEU-4 are collected in all experiments, even though this difference in the results is not significant. This may be due to a difference between the domain of the PHOENIX-2014T dataset and that of the corpus on which GloVe was trained.

The opposite occurs when using pre-trained FastText from Spanish Unannotated Corpora corpus regardless of the size of the training set, producing an improvement of the model with the original configuration without pre-training embeddings over the rest of the pre-trained word embeddings.

### 6.2. Experiment Set 3 (gloss2text Based on ruLSE)

After exploring the performance of Transformer models on the translation to glosses using synthetic dataset tranSPHOENIX, we select the best configurations and analyze them with the more accurate synthetic dataset generated with ruLSE. Table 6 shows a summary of results obtained by selected models with the test set from ruLSE. We can see an improvement in performance as long as we use a larger training set. The best model in all metrics is MarianMT when trained over the whole ruLSE training set. Moreover, both plain STMC model and STMC with pre-trained embedding (FastText from Wikipedia), trained with 7500 sentences, obtain worse results in BLEU-1, BLEU-2 and ROUGE compared to MarianMT trained with only 1000 sentences. Therefore, MarianMT outperforms STMC in all configurations, making it our choice for the following experiment.

It is interesting to illustrate the actual translations (without metrics) performed by the best model, MarianMT, when trained over different subsets from ruLSE. Table 7 shows five samples from ruLSE, with LSE (gloss annotation) and LOE (written Spanish) pairs. The last column shows the generated translation to LOE. We mark in green those that match the original sentence or have the same semantic meaning and a correct syntactic structure, in yellow those sentences that have the same meaning as the reference sentence but do not fully comply with the grammatical rules of the Spanish language, and in red those that do not even share the same meaning. MarianMT trained on 7500 sentences is the only one able to perform translations that are totally correct or very similar to the reference sentence in the selected samples, while the translations generated by the other two models cannot be considered valid. Even so, the 7.5K model produces many errors, so further research is proposed.

So far, we analyzed the models with the test set from the corresponding dataset. Table 8 shows the best model from the previous table (MarianMT trained with 7500 sentences) when tested with valLSE, our small validated sets. As expected, the results are worse when compared with those of Table 6. The results are still acceptable when tested over the *Macarena* set, which consists of 50 sentences generated with ruLSE semi-validated by an interpreter. This is expected since this set uses the same gloss notation to that of the training set of the model. However, we see a drop in performance when testing with the Uvigo set. We highlight two main issues: the gloss notation is still not the same, even though we modify some specific elements to make it more similar to ours; many sentences from the set include only two or three words, which makes it difficult for the model because of the reduced context, and explains the bad results with BLEU-3 and BLEU-4.

Let us recall that these results are not fully comparable with the state of the art in the field, since other related works are based on different datasets (other languages, different gloss codification, other specific use cases, etc.). However, to have a notion of the quality of the performance in general, we report up to 57.61 of BLEU-1, 4.98 BLEU-4 and 46.52 of ROUGE-L over our small semi-validated test set, and 72.33 BLEU-1, 19.9 BLEU-4 and 69.99 ROGUE-L on the test split of ruLSE corpus for the MarianMT model trained on ruLSE. As shown in Table 1, STMC-Transformer [57] (trained on the original RWTH-PHOENIX-Weather 2014T dataset, in German) reports up to 48.41 of BLEU-1, 24.90 BLEU-4 and 48.51 ROUGE-L. Open-NMT (based on LSTMs) [28], trained on Spanish corpus ID/DL and with a synthetic augmentation and pre-training, reports up to 41.63 of BLEU-1 and 45.82 of ROUGE-L. These models are trained on different datasets, so it is not possible to test them on valLSE.

### 6.3. Experiment Set 4 (gloss2text)

We finally focus on SLP and how the models behave when generating gloss annotation instead of written Spanish. We first train the same models as in Experiment Set 2, with the same configurations, on the tranSPHOENIX dataset, inverting the input and object variables. Table 9 reflects a more significant drop in performance for the BLEU metric than for ROUGE, concerning previous experiments, despite using the same amount of data. Therefore, it is confirmed that the used models do not work with the same efficiency for SLP. It is noted that the implementation to train the model without using pre-trained word embeddings provides the best values, which is unusual compared to the results obtained previously. It is also worth noting that, in this case, the MarianMT model does not display, in any case, better performance against any STMC-Transformer configuration.

On the contrary, Table 10 shows that the MarianMT model, when trained on the whole ruLSE dataset (7500 sentences), shows very good behavior for SLP. The best results are obtained on the non-validated test set of ruLSE. We test the performance on the Macarena subset of valLSE (only 50 sentences), whose notation is the same, and the results are also outstanding. This demonstrates that our synthetic corpus generated with the ruLSE system helps to train Transformer models for SLP in an effective way, given that it is more accurate and constructed on top of a set of simple rules.

Of course, the ruLSE gloss generation system is designed for SLP. Therefore, any model trained on the generated dataset would be approaching the developed system. It is expected that the ruLSE system outperforms the best model we can train on it, and so this is shown in Table 11. We highlight that ruLSE is also more efficient in inference time than MarianMT (last column). Therefore, for SLP, we recommend using our system, even though it is not completely accurate.

Finally, to report on a comparison with related works for a notion of performance in text2gloss, Open-NMT [28] (trained on Spanish corpus ID/DL and with a synthetic augmentation and pre-training) reports up to 58.98 BLEU-1 and 73.51 of ROUGE-L.

## 7. Conclusions and Future Work

In this paper, we surveyed the research on automatic Sign Language (SL) communication in both directions: from deaf to hearing and vice versa. First, we analyzed the available SL datasets and their main features. Second, we discussed the different parts in which Sign Language can be treated (recognition, translation and production). And third, we focused on neural Sign Language Translation in both directions with text2gloss and gloss2text, applying a two-Transformer model architecture on a German text dataset translated into Spanish (tranSPHOENIX). It was shown that training smaller datasets does not provide significantly better results, proving the need for a larger dataset to obtain good model performance in translation, similar to the datasets used for typical neural machine translation tasks. It was also concluded that, for the translated dataset employed, the use of pre-trained word embeddings in Spanish increases the performance of the results in most cases, though this increase is not excessively high.

Therefore, we constructed a novel rule-based system named ruLSE to automatically translate glosses from LOE (oral Spanish) to LSE (Spanish Sign Language). This system applies simple rules iteratively, so it is designed to include longer, more complex and complete sentences, providing the model with greater variety. With this system, a large synthetic corpus of 10,000 sentence–gloss pairs taken from the Spanish Speech Text dataset was constructed. We named this the ruLSE dataset. We observed that this more accurate, but still synthetic, dataset improves the training of Transformer models, for both text2gloss and gloss2text. We also verified the results with a validated small dataset with up to 200 pairs in total. It is important to remark on the need for having these data validated by interpreters for a correct transcription of the spoken/written language sentences into glosses. We published all mentioned datasets as a large corpus named *synLSE*.

Thus, as concrete findings after applying the four sets of experiments, we can conclude the following:

1. For the STMC-Transformer model of [57], it was determined that a two-layer encoder–decoder model offers the best results for other layer configurations, as mentioned in the paper.
2. It was shown that the use of a larger data set during training improves the results by approximately four points generically, in both models used. In addition, between smaller training subsets, the most notable performance improvement is 1.15 points (from **15.57** points with a 1000-sentence training to **16.72** for a 3500-sentence training for the BLEU-4 measure), which is not significant considering that more than three times as much data is used during training.
3. The MarianMT model outperformed the Transformer-STMC configurations, but with the trade-off of a significantly longer execution time, about **four times slower**. That is why, despite a 2–3 point improvement of the MarianMT model, the STMC-Transformer model demonstrated higher efficiency, making use of pre-trained word embeddings contributing to superior performance.
4. The use of a more accurate dataset, designed with hand-crafted simple rules to generate glosses from natural language, improved the results for all trained models. MarianMT was still the best model tested in our experiments.
5. The difference between using a translated dataset and a rule-generated dataset increased when applying gloss production. While during SLT tasks, the results for the translated transPHOENIX set improved or worsened slightly, for SLP we could observe a drop of up to **9.14 points** in performance. In contrast, with our synthetic corpus generated with the ruLSE system, there was hardly any drop, so we can state that it is more efficient and accurate for training Transformer models in SLP tasks. Despite its efficiency and accuracy, ruLSE presents a significant trade-off: defining additional rules that add more complexity to the sentence structures requires manual intervention. This complexity contrasts with the ease with which Transformer models can be trained with specific examples.

In future work, we will keep focusing on Sign Language exclusively. Following the branch of Sign Language Translation, we will focus on applying techniques that use keypoint estimation to perform Sign Language Recognition, applying Transformer models to perform translation afterwards. On the other hand, we will launch a SignUS mobile application, in which different user profiles (interpreters, deaf people, individuals who know sign language) will sign a set of simple sentences in written/oral language in order to collect data for the generation of a larger dataset.

As for the ruLSE algorithm, we identified, with the interpreter, some cases that are not covered by the currently defined set of rules. Therefore, as future work, new rules will be created to cover more complex scenarios, errors and sentences recognized by the interpreter to provide the algorithm with greater performance and variety. Here are some examples of such cases:

- The most complex sentences sometimes do not comply with the grammatical structure defined for LSE, but follow another order which consists of signing from the general to the particular to make the message clearer.
- The sign “ahí” (there) has to be added when it is necessary to locate objects in space. Example: “La casa tiene una habitación en la segunda planta donde hay una cama” (”The house has a room on the second floor where there is a bed”) $\overset{LSEglosses}{\to }$ “Casa segunda planta ahí habitación ahí cama”.

## Figures and Tables

**Table 1:** Comparative table of related works performing Sign Language Translation tasks.

Author	Methodology	Dataset	Technique	BLEU-4
Yin et al. (2020) [57]	STMC-Transformer	PHOENIX-2014T	Gloss2Text	24.90
Camgoz et al. (2020) [26]	SLTT	PHOENIX-2014T	Gloss2Text	24.54
Kim et al. (2023) [60]	GRU-based model with keypoint extraction	PHOENIX-2014T	Sign2Text	13.31
McGill et al. (2023) [62]	LSTM attention	iSignos	Gloss2Text	10.22
Chen et al. (2022) [63]	Fully connected MLP with two hidden layers	PHOENIX-2014T	Sign2Text	28.39
Ours (2024)	MarianMT Transformer	SynLSE	Gloss2Text	19.27

**Table 2:** STMC-Transformer model results for text2gloss with the original PHOENIX-2014T dataset in German language. Bold value indicates the best result.

Layers	PHOENIX-2014T Test Set				
	**BLEU-1**	**BLEU-2**	**BLEU-3**	**BLEU-4**	**ROUGE-L**
1	45.66	33.77	26.77	22.15	**46.89**
2	**47.34**	**34.71**	**27.29**	**22.49**	46.51
4	43.39	31.91	25.27	21.13	45.46
6	41.58	30.73	24.51	20.49	44.63

**Table 3:** Results obtained by applying both models with **1000**. Bold value indicates the best result. sentences of the tranSPHOENIX dataset for text2gloss. In *Experiment 2.1*, STMC stands for the configuration of the STMC-Transformer model without using pre-trained word embeddings, STMC + FT(SUC) for the STMC-Transformer model with FastText embeddings from SUC, STMC + G(SBWC) to the STMC-Transformer model with GloVe from SBWC, STMC + FT(Wiki) to the STMC-Transformer model with FastText from Wikipedia and MarianMT for the MarianMT model.

Exper. 2.1	tranSPHOENIX Test Set				
	**BLEU-1**	**BLEU-2**	**BLEU-3**	**BLEU-4**	**ROUGE-L**
STMC	33.79	20.7	18.44	14.11	36.32
STMC + FT(SUC)	33.44	24.61	18.64	14.45	37.27
STMC + G(SBWC)	33.66	25.11	18.03	13.88	37.07
STMC + FT(Wiki)	33.73	24.47	18.34	13.87	37.81
MarianMT	**38.58**	**27.78**	**20.51**	**15.57**	**38.98**

**Table 4:** Results obtained by applying both models with half (**3500 sentences**) of the tranSPHOENIX dataset for text2gloss. In the *Experiment 2.2* column, STMC stands for the configuration of the STMC-Transformer model without using pre-trained word embeddings, STMC + FT(SUC) for the STMC-Transformer model with FastText embeddings from SUC, STMC + G(SBWC) to the STMC-Transformer model with GloVe from SBWC, STMC + FT(Wiki) to the STMC-Transformer model with FastText from Wikipedia and MarianMT for the MarianMT model. Bold value indicates the best result.

Exper. 2.2	tranSPHOENIX Test Set				
	**BLEU-1**	**BLEU-2**	**BLEU-3**	**BLEU-4**	**ROUGE-L**
STMC	35.70	25.74	19.5	15.37	38.01
FT + SUC	36.84	26.7	20.33	15.9	38.94
G + SBWC	33.45	24.42	18.47	14.5	37.88
FT + Wiki	37.68	27.11	**20.86**	15.86	**38.96**
MarianMT	**39.45**	**28.25**	20.8	**16.72**	34.85

**Table 5:** Results obtained by training both models on the entire tranSPHOENIX dataset for text2gloss. In the *Experiment 2.3* column, STMC stands for the configuration of the STMC-Transformer model without using pre-trained word embeddings, STMC + FT(SUC) for the STMC-Transformer model with FastText embeddings from SUC, STMC + G(SBWC) to the STMC-Transformer model with GloVe from SBWC, STMC + FT(Wiki) to the STMC-Transformer model with FastText from Wikipedia and MarianMT for the MarianMT model. Bold value indicates the best result.

Exper. 2.3	tranSPHOENIX Test Set				
	**BLEU-1**	**BLEU-2**	**BLEU-3**	**BLEU-4**	**ROUGE-L**
STMC	39.13	29.36	22.8	18.31	42.8
FT + SUC	41.63	30.95	23.76	18.87	43.21
G + SBWC	35.53	26.91	20.85	16.98	42.01
FT + Wiki	39.43	29.57	22.91	18.37	42.61
MarianMT	**42.89**	**31.85**	**24.45**	**19.27**	**43.91**

**Table 6:** Results obtained by training both models on the ruLSE dataset for text2gloss. In the *Experiment 3.1* column, MarianMT (x) stands for the configuration of the MarianMT model trained on x sentences from the ruLSE train set (for x being 1000, 3500 and 7500). STMC stands for the configuration of the STMC-Transformer model without using pre-trained word embeddings and STMC + FT(Wiki) to the STMC-Transformer model with FastText from Wikipedia. Bold value indicates the best result.

Exper. 3.1	ruLSE Test Set				
	**BLEU-1**	**BLEU-2**	**BLEU-3**	**BLEU-4**	**ROUGE-L**
MarianMT (1000)	55.42	26.54	14.13	9.29	51.8
MarianMT (3500)	67.24	37.39	22.4	14.49	61.44
MarianMT (7500)	**72.33**	**44.01**	**28.8**	**19.9**	**69.99**
STMC (7500)	44.22	28.89	18.49	11.94	39.57
FT + Wiki (7500)	38.78	25.49	16.56	10.89	38.21

**Table 7:** Example text2gloss translations performed by the MarianMT model trained over different subsets of the ruLSE dataset. Literal English translations are in italics under each sentence. In the LOE (generated) column we differentiate green background for correct translations, yellow for intermediate translations and red for incorrect translations.

MarianMT Model	LSE (Input)	LOE (Original)	LOE (Generated)
Trained with 1000	PASADO PEPE-NP COCHE COMPRAR PEPA-MP *(PAST PEPE CAR BUY PEPA)*	Pepe compró un coche a Pepa *(Pepe bought a car from Pepa)*	compró pepa y pepe coche *(bought pepa and pepe car)*
Trained with 3500			pepe coche compró pepa *(pepe car bought pepa)*
Trained with 7500			pepe compró un coche a pepa *(pepe bought a car from pepa)*
Trained with 1000	JESÚS-NP CAMISA SUCIO MUCHO LLEVAR *(JESUS DIRTY SHIRT MUCH WEAR)*	Jesús lleva la camisa muy sucia *(Jesus is wearing a very dirty shirt)*	lleva desde la casa de el sucio hasta muy lejos *(leads from his dirty house all the way to far away)*
Trained with 3500			jesús lleva una camisa de mucho sucio *(jesus wears a very dirty shirt)*
Trained with 7500			jesús lleva una camisa muy sucia *(jesus is wearing a very dirty shirt)*
Trained with 1000	PELÍCULA APTO MENOR *(FILM SUITABLE FOR MINORS)*	La película es apta para menores *(The film is suitable for minors)*	la película es apto para el menor *(the film is suitable for children)*
Trained with 3500			la película es apta para ser menor *(the fil is suitable for minors)*
Trained with 7500			película apta para ser menor *(film suitable for minors)*
Trained with 1000	PREMIO DAR QUIÉN *(AWARD GIVE WHO)*	¿A quién han dado el premio? *(To whom did they give the award?)*	dar a quién el premio *(give to whom the award)*
Trained with 3500			el premio da a quien sea *(the award goes to anyone)*
Trained with 7500			el premio fue dado a quién *(the award was given to whom)*
Trained with 1000	CUADRO ESTE PINTAR ARTISTA MAGNÍFICO *(PICTURE THIS PAINTING MAGNIFICENT ARTIST)*	Este cuadro ha sido pintado por el magnífico artists *(This picture has been painted by the magnificent artist)*	este cuadros pintaba en la artists magnífica *(this painting was painted in the magnificent artists)*
Trained with 3500			este cuadro pinta y es un artists magnífico *(this picture paints and is a magnificent artist)*
Trained with 7500			este cuadro fue pintado por el artists magnífico *(this picture was painted by the magnificent artist)*

**Table 8:** Results of applying the MarianMT model trained on valLSE for text2gloss, our semi-validated sets in the SynLSE corpus. Bold value indicates the best result.

ValLSE	MarianMT Model				
	**BLEU-1**	**BLEU-2**	**BLEU-3**	**BLEU-4**	**ROUGE-L**
Macarena (50)	**57.61**	**23.21**	**9.98**	**4.98**	**46.52**
Uvigo (150)	28.46	5.87	1.04	0.56	36.08

**Table 9:** Results of applying the MarianMT model and STMC-Transformer with different word embeddings by training both models in gloss2text, i.e., from sentences in natural language to annotated glosses (a former step towards SLP) on tranSPHOENIX. Bold value indicates the best result.

Configuration	tranSPHOENIX Test Set				
	**BLEU-1**	**BLEU-2**	**BLEU-3**	**BLEU-4**	**ROUGE-L**
STMC	**37.5**	**22.36**	**14.65**	**10.13**	**37.94**
FT + SUC	35.83	20.83	13.45	9.32	37.61
G + BWC	33.15	19.09	12.26	8.52	35.95
FT + Wiki	36.43	21.04	13.42	9.15	37.27
MarianMT	21.55	10.89	5.91	3.46	30.24

**Table 10:** Results of the MarianMT model trained on 7500 sentences from ruLSE on the ruLSE test set and on the Macarena subset (50 sentences) of valLSE for gloss2text.

Dataset	MarianMT Model on ruLSE Set				
	**BLEU-1**	**BLEU-2**	**BLEU-3**	**BLEU-4**	**ROUGE-L**
ruLSE (test)	90.61	72.84	61.15	52.37	87.67
Macarena	74.08	39.63	22.71	12.07	58.84

**Table 11:** Results of the MarianMT model trained on 7500 sentences from ruLSE versus the ruLSE gloss generation system over the whole valLSE dataset (Macarena and Uvigo) for gloss2text. Bold value indicates the best result.

	MarianMT and ruLSE System on valLSE					
	**BLEU-1**	**BLEU-2**	**BLEU-3**	**BLEU-4**	**ROUGE-L**	**Time (s)**
ruLSE system	**70.33**	**37.21**	**28.39**	**21.05**	**60.53**	**0.3011**
MarianMT (text2gloss)	53.75	17.33	8.57	4.61	50.83	1.1308

## Data Availability

The dataset generated and employed in this research, along with the associated source code, are available at https://github.com/Deepknowledge-US/TAL-IA/tree/main/CORPUS-SynLSE (accessed on 20 February 2024), as part of the TAL.IA project https://github.com/Deepknowledge-US/TAL-IA (accessed on 20 February 2024).
